# Preparation and Evaluation of the Effectiveness of an Anti‐Wrinkle Cream Containing *Equisetum arvense* and Soybean Extracts

**DOI:** 10.1111/jocd.16734

**Published:** 2024-12-27

**Authors:** Sara Nojomi, Fatemeh Soltanmohammadi, Hamideh Azimi Alamdari, Sanaz Hamedyazdan, Adel Mahmoudi Gharehbaba, Yousef Javadzadeh

**Affiliations:** ^1^ Department of Pharmaceutics, Faculty of Pharmacy Tabriz University of Medical Sciences Tabriz Iran; ^2^ Department of Dermatology, Faculty of Medicine Tabriz University of Medical Sciences Tabriz Iran; ^3^ Department of Pharmacognosy, Faculty of Pharmacy Tabriz University of Medical Sciences Tabriz Iran

**Keywords:** anti‐aging, anti‐wrinkle, forehead wrinkles, horsetail, soybean

## Abstract

**Background:**

Skin aging is a multifaceted condition marked by the development of wrinkles, reduced suppleness, and uneven pigmentation. Both endogenous and exogenous factors contribute to skin aging. Studies have examined the possible anti‐aging advantages of horsetail and soybean extracts, which are abundant in antioxidants.

**Methods:**

A study was conducted to develop and assess the stability of an anti‐wrinkle cream combining horsetail and soybean extracts over a period of 6 months. This study was a pilot study that was prospective and noncomparative in nature. An investigation was performed to explore the efficiency of a treatment on wrinkles on the forehead. The study involved 15 volunteers between the ages of 35 and 55, and the modified Fitzpatrick wrinkle grading scale (FWS) method was used to assess the results. The participants were directed to administer cream to the forehead region for a duration of eight sequential weeks. Images were acquired at the initial stage and after 8 weeks of treatment. An independent investigator evaluated the improvement in the overall appearance of the forehead skin by comparing clinical images taken before and after the therapy.

**Results:**

The cream exhibited exceptional stability, showing no noteworthy alterations in pH, viscosity, or microbiological count. The clinical findings demonstrated a notable decrease in the average FWS score from 8.7 ± 1.3 to 6.1 ± 1.8 (*p* < 0.005) after the treatment. 93.4% of the individuals experienced a positive alteration in the appearance of their forehead wrinkles.

**Conclusion:**

The formulated cream presents a hopeful method to tackle skin aging issues, backed by its durability and effectiveness in diminishing wrinkles. Additional investigation is necessary to clarify the underlying mechanisms.

**Trial Registration:** IRCT20190917044799N1

## 1 Introduction

**FIGURE 1:**
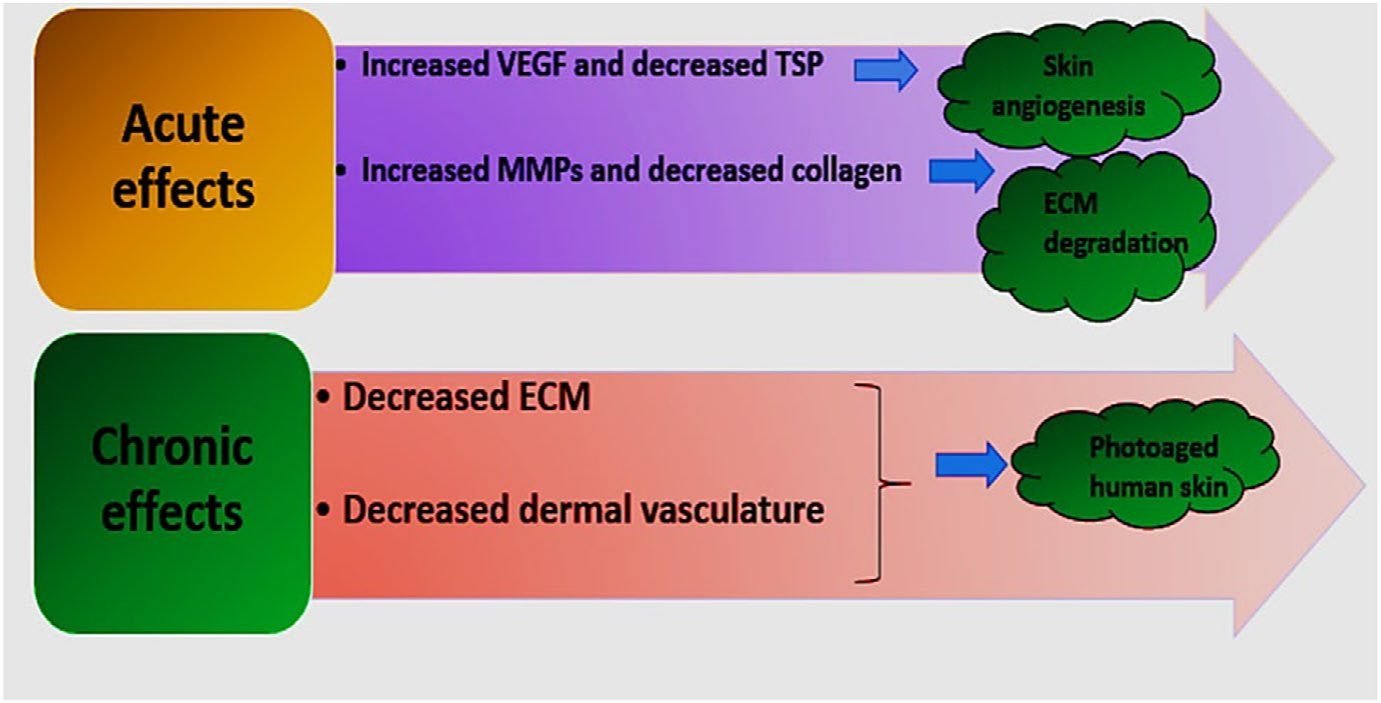
Acute and chronic effects of UV on skin (extracellular matrix (ECM), vascular endothelial growth factor (VEGF), matrix metalloproteinase (MMP), thrombospondin (TSP)).

Skin aging, a prevalent dermatologic occurrence, encompasses two distinct processes. Chronological aging, also known as intrinsic aging, occurs naturally over time and is evident in the skin of elderly individuals, even if they have taken measures to protect it from the sun. In contrast, premature aging, also known as photo (extrinsic) aging, is a rapid process that results from environmental factors such as excessive exposure to ultraviolet (UV) light, smoking, solar radiation, and poor lifestyle choices [1, 2]. Exposure to UV irradiation is the key factor responsible for extrinsic aging. Visible indications of photoaging include the development of wrinkles, sagging skin, loss of elasticity, excessive pigmentation, dilated blood vessels, and delayed healing of the skin [3, 4]. Figure 1 illustrates the long‐term and immediate consequences of being exposed to UV radiation.

In order to combat the effects of skin aging and prevent its associated consequences, it is crucial to develop more effective skincare products. The horsetail plant (*Equisetum arvense*) is a perennial, nonflowering plant characterized by black rhizomes and two types of stems: spring and summer stems. This plant contains vitamin C, vitamin E, zinc, and copper, which contribute to its ability to scavenge reactive oxygen species (ROS) through the action of superoxide dismutase [5]. It is important to mention that the radical scavenging effect is directly related to the phenolic content of the extract. Additionally, the plant acts as a hyaluronidase inhibitor, which prevents the breakdown of hyaluronic acid and allows it to remain in its polymeric state. This enables hyaluronic acid to serve as a crucial agent for development, growth, and tissue repair [6]. Soybean ( *Glycine max* ) is a medicinal plant recognized for its potent antioxidant properties, attributed to its high content of phenolic compounds, including various isoflavones and their conjugates with glucosides [7, 8]. Soybeans possess the ability to eliminate harmful free radicals generated by exposure to UV radiation, thanks to the antioxidant properties of isoflavones. Additionally, soybeans may effectively penetrate the skin due to their content of conjugated isoflavones [9, 10, 11]. Aside from its antioxidant effects, vitamin E found in soybeans is able to hydrate the skin, promoting skin renewal and reducing the appearance of wrinkles. Other constituents in soybeans also support collagen production and enhance skin elasticity [12]. In this study, the anti‐wrinkle impact of the topical cream containing horsetail and soybean extract was evaluated.

## 2 Materials and Methods

### 2.1 Materials

Flowers of the horsetail plant were collected in spring, especially in late May, from the mountains and plains of East Azerbaijan province. They were identified at the herbarium of the Faculty of Pharmacy. Soybeans were purchased from Merck (Germany). Eucerin, propyl paraben, and methyl paraben were purchased from Merck (Germany).

### 2.2 Preparation of Ointment

The hydroalcoholic extract of each plant was attained using high‐analytical grade pharmaceutical excipients. Total extraction was performed by soaking the plant materials in a water and ethanol mixture in a ratio of 30:70. The achieved hydroalcoholic extract was dried in a rotary evaporator, yielding a brown powder that was utilized in the ointment formulation. To formulate the powders obtained from soybean and horsetail, the following procedure was briefly performed:

- 2 g of the extract powder was added to 20 mL of hydroalcoholic solvent containing 14 mL of ethanol and 6 mL of water, and the powder was dissolved in the solvent. Finally, a clear brown solution was obtained. The resulting solution was transferred to a beaker. Then a magnet and heater were used for controlled stirring. For larger‐scale preparation in the laboratory, an industrial stirrer was utilized to create the formulation.
- 15 g of Eucerin was added to the solution.
- Methyl paraben and propyl paraben were added in 0.2% and 0.02% weight ratios, respectively.
- At this stage, a cream base prepared from Sepidaj factory was added to the formulation.

### 2.3 Stability Evaluation

The cream was subjected to accelerated stability testing, where it was stored at a temperature of 40°C ± 2°C and a relative humidity of 75% ± 5% for a duration of 6 months. A comprehensive physicochemical examination, encompassing color, odor, homogeneity, pH, viscosity, density, and microbiological evaluations, was conducted at months 0, 1, 2, 4, and 6 (Tables 1 and 2).

**TABLE 1:** Stability evaluation results.

Test	Initial	First month	Second month	Fourth month	Sixth month	Standard
Description	Meets standard	Meets standard	Meets standard	Meets standard	Meets standard	Brown and homogenous cream
Odor	Meets standard	Meets standard	Meets standard	Meets standard	Meets standard	Characteristic order
pH	6.4	6.4	6.5	6.7	6.7	NLT 5.00 NMT 7.00
Density (g/cm^3^)	0.991	0.991	0.992	0.992	0.992	NLT 0.980 NMT 0.999
Viscosity (cP)	14 235	14 239	14 291	1552	1576	NLT 13000 NMT 17000

**TABLE 2:** Antimicrobial test results.

Test	Initial	First month	Second month	Fourth month	Sixth month	Standard
Total bacterial count (CFU/g)	1	1	0	1	2	NMT 100
Total fungi and yeast count (CFU/g)	1	1	0	0	0	NMT 10
*Pseudomonas aeruginosa*	Negative	Negative	Negative	Negative	Negative	Must be negative
*Staphylococcus aureus*	Negative	Negative	Negative	Negative	Negative	Must be negative
Antimicrobial effectiveness test	Ok	Ok	Ok	Ok	Ok	Bacteria: A minimum reduction of 2 log from the initial count is required at 14 days, with no enhancement in the count from day 14 to day 28 Yeast and Mold: The count should not exceed the initial calculated value at both 14 and 28 days

### 2.4 Study Design

A prospective, noncomparative pilot study was conducted over a period of 8 weeks to assess the antiaging properties of a cream that contains horsetail and soybean extract on facial skin. The investigation was conducted at the dermatological clinic of Sina Hospital, with trial registration number (IRCT20190917044799N1). This study was endorsed by the Ethics Committee (IR.TBZMED.REC.1397.1027). Furthermore, the study was carried out in accordance with the ethical guidelines outlined in the Declaration of Helsinki and Good Clinical Practice (GCP). The study methodology was presented to all participants, followed by the acquisition of written informed consent. Participants were instructed to apply a single unit of the cream on their forehead once daily for a duration of 8 weeks. Both baseline and 8‐week measurements were conducted using noninvasive methods.

### 2.5 Participants

A total of fifteen individuals who were in good health were selected to take part in the study.

Participants were categorized according to the modified FWS [13].

All individuals had forehead wrinkles, and the modified FWS scores varied from 1 to 12 (Table 3).

**TABLE 3:** Modified Fitzpatrick wrinkle grading scale.

Grade	Score	Wrinkling	Degree of elastosis
0	1–3	Absence of wrinkles	—
0.5	4–6	Very shallow yet visible wrinkle, moderate number of lines	Mild (the skin exhibits mild textural changes with minor skin wrinkles)
1.0	7–9	Visible wrinkle with slight indentation	Moderate (the condition is characterized by moderate levels of distinct popular elastosis, individual papules that have a yellow translucency, and dyschromia)
1.5	10–12	Visible wrinkle and clear indentation	Severe (the individual exhibits severe symptoms including the presence of many papules and confluent elastosis, as well as thickened yellow and pallid cutis rhomboidalis)

### 2.6 Inclusion and Exclusion Criteria

The following circumstances were established as the criteria for inclusion.

1. Female participants who were in good health, aged between 35 and 55.
2. Exhibited symptoms of wrinkles.
3. Subjects are expected to adhere to a written guideline provided by the team and comply with the guidelines regarding their habits, such as food, physical activity, usage of makeup, facial cosmetics, cleansing products, and sunglasses.

The criteria for exclusion were as follows:

1. Utilizing any systemic or topical therapies specifically designed to reduce wrinkles or any procedures aimed at slowing down the aging process, as well as injectable fillers, during the past year.
2. Recent usage of isotretinoin within the last 3 months.
3. Medical procedures (such as thorough chemical peeling or laser therapy) within the last 6 months.
4. Existence of cystic or severe acne on the face.
5. Smoking history in the 24 months preceding the study.
6. Women who are pregnant or are breastfeeding.

### 2.7 Evaluations of Enhancement in the Forehead Skin by Participants and Independent Investigator

**FIGURE 2:**
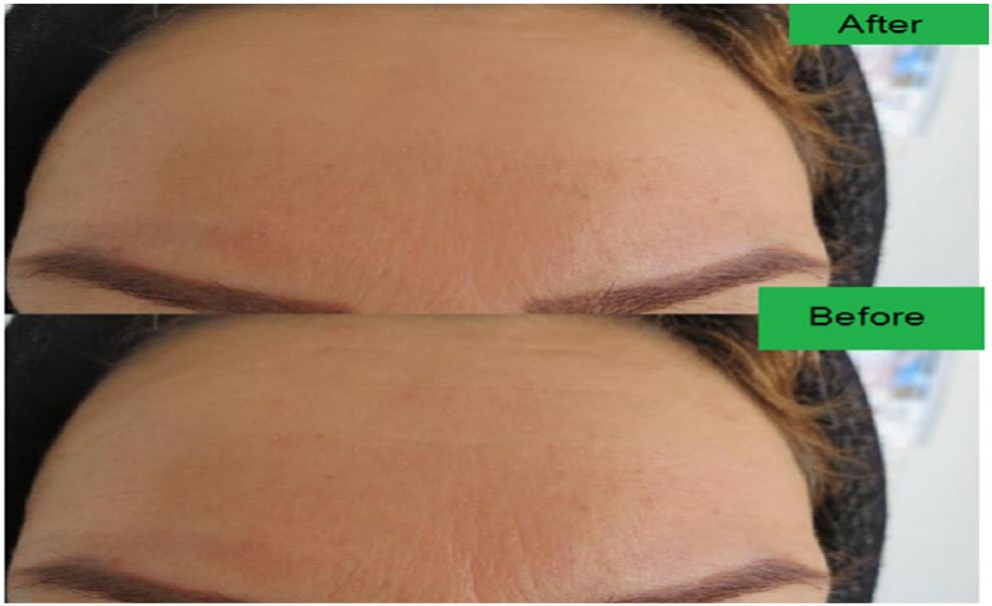
Formulation effect on forehead wrinkles removal.

Photographs were taken at the initial stage and after 8 weeks of therapy with the formulation (Figure 2). The clinical response to treatment was evaluated by an independent investigator, who compared clinical images taken before and after treatment, along with the subjective improvement scores reported by the participants. An independent investigator analyzed baseline and postapplication images by the modified FWS system and quartile score. The investigator specifically examined the circumstance of the forehead skin for each individual. The revised FWS system was evaluated using a numerical scale ranging from 1 to 12, as depicted in Table 3. The investigator evaluated the quartile score using a scale ranging from 1 to 4. A score of 1 indicated less than 25% improvement, a score of 2 indicated 26% to 50% improvement, a score of 3 indicated 51% to 75% improvement, and a score of 4 indicated more than 75% improvement. The average of the modified FWS scores for all participants was subsequently computed for both the baseline and postapplication photos. Participants were asked to rate their subjective progress and satisfaction with the treatment using a 5‐point Likert scale. A score of 0 showed a worse appearance, 1 showed no improvement, 2 showed fair improvement, 3 showed good improvement, and 4 showed great improvement (Tables 5 and 6).

### 2.8 Safety Evaluation

Participants underwent examinations at the beginning of the study and at 1, 2, 4, and 8 weeks to assess any negative reactions, including inflammation, scaling, and subjective sensory discomforts (itching, burning, and stinging) (Table 4). If a participant encountered a profound localized skin irritation, the treatment was halted [14].

**TABLE 4:** Subject characteristics and reported side effects.

Subject	Age	Skin phenotype grade	Side effect
1	45	1	No
2	43	1	No
3	46	1	Itching
4	49	1.5	No
5	52	1.5	Irritation
6	55	1.5	No
7	54	1.5	No
8	53	1.5	Itching
9	50	1.5	No
10	35	0.5	No
11	37	0.5	No
12	48	1	No
13	51	1.5	Irritation
14	53	1.5	No
15	43	1	Itching

### 2.9 Statistical Analysis

The statistical analysis was conducted using IBM SPSS Statistics software version 20, developed by IBM Corporation. Percentage (%) was utilized to represent quantitative data, while mean value and standard deviation (SD) were employed for qualitative data frequency. To compare the baseline with each treatment value, a paired *t*‐test or Wilcoxon test was used. Only data with a *p*‐value less than 0.05 were considered significant.

## 3 Results

This study aimed to develop a cosmetic cream with acceptable stability using an adequate analysis method. The topical anti‐wrinkle cream successfully underwent accelerated stability testing for a duration of 6 months, as indicated in Table 1. The freshly prepared cream displayed a uniform texture and a brown hue. Throughout the storage period at a temperature of 40°C ± 2°C and a relative humidity of 75% ± 5%, the formulation exhibited no alterations in color, odor, pH, or homogeneity (absence of phase separation).

The average pH levels at the start, 1st, 2nd, 4th, and 6th months were 6.4, 6.4, 6.5, 6.7, and 6.7, respectively. The viscosity measurements at the initial, 1st, 2nd, 4th, and 6th months were 14 235, 14 239, 14 291, 1552, and 1576 cP, respectively. The density of fresh cream was 0.991 at month 1, and 0.991, 0.992, 0.992, and 0.992 at months 2, 4, and 6, respectively. The microbiological evaluation conducted at the initial, 1st, 2nd, 4th, and 6th months revealed the presence of 1, 1, 0, 1, and 2 bacterial colonies, respectively. The fungal counts were 1, 1, 0, 0, and 0 colonies. These results show that the antimicrobial activity has been sufficient and effective. The results were enumerated in Table 2.

The demographic characteristics of the subject are displayed in Table 3. The study was completed by all 15 healthy participants, who were between the ages of 35 and 55. The participants had a median age of 47 ± 3.6 years, and they were all female. The majority of the participants exhibited skin phenotypes 1 and 1.5, accounting for 86.9% of the total (Table 4). During the post‐treatment evaluation, 93.4% of the subjects reported a “fair” or “good” improvement in their forehead wrinkles, according to a 5‐point Likert scale. Only 6.6% of the subjects reported no change. None of the participants reported an exacerbation of their wrinkles (Table 5). According to the modified FWS system, there was a noticeable decrease in the participants' score from the initial assessment to the assessment after treatment (8.7 [mean] ±1.3 [SD] vs. 6.1 ± 1.8 [*p* < 0.005]); (Table 6). Out of the five participants, none of them stopped using the formulation despite experiencing modest adverse effects such as irritation and itching (Table 4).

**TABLE 5:** Descriptive statistics.

Characteristic	*N* (%)
Age (years) mean ± SD [range]	47 ± 3.6 [35–55]
Skin phenotype	
0	0 (0)
0.5	2 (13.3)
1	5 (33.3)
1.5	8 (53.3)
% of improvement as assessed by patient^a^	
0‐Worse	0 (0)
1‐No change	1 (6.6)
2‐Fair	5 (33.3)
3‐Good	9 (60.1)
4‐Excellent	0 (0)
% of improvement evaluated by investigator^b^	
1‐≤ 25%	3 (20.0)
2‐26%‐50%	6 (40.0)
3‐51%‐75%	6 (40.0)
4‐> 75%	0 (0)
Adverse effects	
Irritation	2 (13.3)
Itching	3 (20.0)

^a^ 5‐point Likert scale; assessed by the subjects where 0 = worse, 1 = none, 2 = fair, 3 = good, 4 = excellent.

^b^ The investigator evaluated the quartile score using a scale ranging from 1 to 4. A score of 1 indicated less than 25% improvement, a score of 2 indicated 26% to 50% improvement, a score of 3 indicated 51% to 75% improvement, and a score of 4 indicated more than 75% improvement.

**TABLE 6:** A comparative analysis of pre‐ and post‐treatment outcomes utilizing the modified Fitzpatrick wrinkle‐scoring system.

	Before	After	*p*
Investigator, mean ± SD (range)	8.7 ± 1.3 (4–12)	6.1 ± 1.8 (4–9)	> 0.005

## 4 Discussion

Intrinsic aging is characterized by the thinning of the skin's dermal and epidermal layers, a decrease in the number of mast cells and fibroblasts, a loss in collagen, and an increase in matrix metalloproteinase (MMP) levels [15, 16]. Extrinsic aging, on the other hand, results from prolonged exposure to UV radiation from the sun or other environmental causes. Extended exposure to sunlight induces alterations in the extracellular matrix (ECM), resulting in the development of elastic tissue on the skin's surface. This elastic tissue is marked by changes in skin thickness and the production of wrinkles [17]. Moreover, this extended duration of exposure triggers signaling pathways that result in the production of MMP genes, leading to the deactivation of collagen and elastin. UV radiation induces the synthesis of certain transcriptional factors. The synthesis of these factors stimulates the generation of the cytokines. Cytokines are pro‐inflammatory agents that trigger inflammation and stimulate the immune system [4].

The formulated topical cream exhibited appropriate sensory qualities and stability. No changes in color, odor, or pH were seen during accelerated stability testing, and the texture remained consistent. The microbiological examination confirms that the preservation activity of parabens has been sufficient and effective (Tables 1 and 2).

All 15 volunteers successfully completed the study and reported a high level of adherence to the provided instructions. Noninvasive skin measures were utilized in the computation of skin conditions.

After a duration of 8 weeks, the examination of the ranking image revealed that the utilization of the topical anti‐wrinkle cream resulted in a substantial enhancement in volume and area of wrinkles, as compared to the initial values (Tables 5 and 6). The reduction in wrinkles can be attributed to the presence of powerful antioxidant components, silica, and saponin in the plant extract, as well as its capacity to block hyaluronidase. The effectiveness of soybean in improving skin wrinkles may be attributed to its proteases, abundant vitamin E content, and antioxidants. The utilization of various components, such as UV‐induced free radicals, restored laminin, elastic fiber, and collagen structure, promoted the generation of collagen and effectively decreased the breakdown of collagen and hyaluronic acid.

In a similar study, Saad Altalhab [14] assessed the efficacy of imiquimod 5% cream as a treatment for wrinkles. The results indicated that 72.7% of the participants observed a noticeable improvement in periorbital skin changes, as assessed by a 5‐point Likert scale. In our study, 93.4% of the subjects reported a “fair” or “good” improvement in their forehead wrinkles. The investigator also evaluated the quartile score, which revealed that seven subjects (63.6%) showed a minimum of 26% improvement. In our study, we found that 80% of the participants showed a minimum of 26% improvement. Ozay et al. developed a topical medication using an extract from the *Equisetum arvense* plant, which effectively stimulated the growth of skin, the outermost layer of skin, and tissue granules in mice after 60 days of application [18]. Horsetail extract has been shown to interact with immune cells and possess an anti‐inflammatory effect in a separate study [19]. Another documented alteration in the process of skin aging is the development of skin pigmentation. Paine and his colleagues demonstrated that proteases found in soybean and soy milk effectively removed pigmentation in keratinocytes, both in vitro and in vivo [20]. In a separate research, the direct application of soy extract onto the skin of women who had prolonged exposure to sunshine resulted in skin lightening [21]. A study has demonstrated that the utilization of soy phytosterols in conjunction with other components yields a more pronounced impact on enhancing erythema in patients [22]. Hence, in this investigation, we employed a blend of soybean and horsetail extract to enhance skin wrinkles that may arise from either inherent or environmental aging.

The physicians and patients subjectively evaluated the improvement of skin wrinkles compared to the baseline. An assessment of skin aging was conducted by a dermatologist via the modified FWS technique to evaluate wrinkle grading. The results indicated improvement in skin aging following the treatment period. Furthermore, the Likert scale indicated that individuals experienced a level of satisfaction that might be described as “moderate” upon completion of the treatment. There were no significant negative effects seen, and no instances of skin redness, burning, or peeling were reported during the treatment (Table 4).

## 5 Conclusion

The preparation, evaluation, and in vivo evaluation of the effectiveness of an anti‐wrinkle cosmetic cream in which horsetail and soybean were combined were the objectives of this study. Under the conditions of accelerated stability studies (40°C ± 2°C/75% ± 5% RH), the composition of extracts and the base of cream remain stable for a period of up to 6 months. The application of this cream on a daily basis was not only risk‐free, but it also provides effective renewal of facial skin by decreasing the visible signs of aging. It is important to note that this study was not a split‐face study, which may limit the ability to directly compare the effects of the cream on distinct areas of the skin and account for genetic variations among the participants. Additionally, the absence of digital imaging analysis restricts the quantitative assessment of changes in skin texture and appearance. Future studies should consider these factors to provide a more complete evaluation of the effectiveness of the cream.

## Author Contributions

**Sara Nojomi:** writing – original draft. **Fatemeh Soltanmohammadi:** writing – review and editing. **Adel Mahmoudi Gharehbaba:** writing – review and editing. **Sanaz Hamedyazdan:** supervision, writing – review and editing. **Hamideh Azimi Alamdari:** conceptualization, supervision, writing – review and editing. **Yousef Javadzadeh:** conceptualization, supervision, writing – review and editing. All authors have read the journal's authorship agreement and the manuscript has been reviewed by and approved by all named authors.

## Ethics Statement

The proposal and consent form were approved by the ethical committee of the Faculty of Pharmacy at Tabriz University of Medical Science (IR.TBZMED.REC.1397.1027).

## Consent

The authors have nothing to report.

## Conflicts of Interest

The authors declare no conflicts of interest.

## Data Availability

All data generated or analyzed during this study are included in this published article.
